# Long-Term Organic Amendment Systems Are Associated with Pore–Aggregate Structure, Root Traits, and Labile Organic Carbon Allocation in a Brown Soil

**DOI:** 10.3390/plants15172562

**Published:** 2026-08-23

**Authors:** Hairui Ma, Xiao Li, Shuanglong Yang, Ni Zhang, Xinyu Mu, Shunguo Liu, Xiumei Zhan

**Affiliations:** 1 College of Land and Environment, Shenyang Agricultural University, Shenyang 110866, China; 2025220500@stu.syau.edu.cn (H.M.); 13192708513@163.com (X.L.); yangsl0830@163.com (S.Y.); zn903164341@163.com (N.Z.); 18347568221@163.com (X.M.); 2 Liaoning Agricultural and Rural Development Service Center, Shenyang 110034, China; lnsspb@163.com

**Keywords:** organic amendments, soil pore structure, soil aggregates, labile carbon fractions, peanut root morphology, X-ray computed tomography

## Abstract

Organic amendments can alter soil structure, root development, and carbon cycling, yet their coordinated effects remain unclear. Based on a long-term field microplot experiment established in 2009, four amendment systems with equivalent annual N, P, and K inputs but differing in amendment properties and nominal annual organic-material C inputs were compared: maize straw with NPK (CS), pig manure compost with NPK (PMC), biochar with NPK (BIO), and biochar-based fertilizer (BF). After 15 years, dry-sieved aggregate distribution, CT-resolved air-filled pores (>30 μm), peanut root morphology, and easily oxidizable organic carbon (EOC), microbial biomass carbon (MBC), and dissolved organic carbon (DOC) were determined. PMC had the highest CT-resolved total and connected porosities (19.01% and 10.86%), a greater proportion of small macroaggregates, and the largest root surface area. CS produced a greater proportion and mean size of large dry-sieved aggregates and the highest bulk-soil MBC content. BIO and BF showed lower CT-resolved total porosity but greater isolated porosity, anisotropy, mean pore diameter, and pore fractal dimension (collectively termed CT-resolved macropore heterogeneity); these treatments were also associated with greater root volume or length and increased EOC and DOC contents in small macroaggregate- and microaggregate-sized fractions. Root length correlated more strongly with macropore heterogeneity than with total porosity. Because measurements were obtained once from 12 microplots, these relationships and SEM results represent exploratory associations rather than causal pathways. Overall, traditional amendments were associated with aggregation or macropore connectivity, whereas carbonized amendments were associated with greater macropore heterogeneity. BF had the highest percentage of EOC in TOC (52.45%), indicating a greater relative contribution of labile carbon, not increased stable carbon stock.

## 1. Introduction

Soil structural conditions strongly influence root exploration, resource acquisition, and belowground carbon cycling in agroecosystems [1,2]. Organic amendments can modify aggregate formation and pore architecture, thereby changing the physical space available for root elongation and the accessibility of labile carbon to microorganisms [3,4]. However, amendment-induced increases in aggregate size do not necessarily coincide with greater pore connectivity, and total porosity alone may not adequately explain root responses. A combined assessment of aggregates, three-dimensional pore geometry, root morphology, and labile carbon allocation is therefore required to clarify how long-term amendment systems reshape plant–soil interactions [5,6].

Maize straw and pig manure are categorized as conventional organic amendments and generally provide relatively high inputs of organic carbon and biologically available substrates [7,8,9]. Maize straw has a high C/N ratio and retains the distinct physical framework of plant residues. After incorporation into soil, straw residues and their decomposition products may participate in aggregate formation through particulate organic matter accumulation, microbial binding agents, and fungal hyphal entanglement [10,11,12]. In contrast, pig manure has a relatively low C/N ratio and higher nutrient availability, making its organic components more readily utilized by soil microorganisms. This may stimulate microbial metabolism and the production of organic binding agents, thereby affecting soil particle aggregation and pore-network development [7,13]. Biochar and biochar-based fertilizers are pyrolyzed organic amendments characterized by high aromaticity, porous particle structures, and relatively large specific surface areas [14,15]. Owing to their comparatively recalcitrant carbon fractions and distinct particle properties, these amendments may alter soil aggregation and pore geometry through particle filling, surface adsorption, and biochar–mineral interactions [16,17]. Because these amendment systems also differ in annual carbon input and nutrient-release patterns, their long-term effects should be interpreted as contrasts among complete fertilization systems rather than as isolated effects of amendment type alone.

Soil pores and aggregates serve as vital spatial vehicles linking organic matter inputs with soil carbon transformation [18,19,20]. Distinct pore architectures can influence nutrient transport and root development, whereas aggregate-size fractions differ in the enrichment and accessibility of organic carbon [6,18,20]. Labile organic carbon fractions, such as easily oxidized organic carbon (EOC), microbial biomass carbon (MBC), and dissolved organic carbon (DOC), respond sensitively to soil management practices, effectively reflecting soil carbon pool turnover dynamics and nutrient supply potential [21,22]. Compared with bulk soil SOC, the distribution of labile carbon fractions within different aggregate size classes provides a clearer depiction of the spatial positioning and potential transformation pathways of organic carbon within the soil matrix [23,24]. Therefore, evaluating the impacts of organic amendments on soil carbon pool quality should extend beyond changes in bulk soil carbon content alone, warranting a closer examination of associations between pore–aggregate architecture and the spatial allocation of labile carbon fractions [25,26].

Roots represent a crucial nexus connecting soil structure with carbon cycling processes. On the one hand, pore size, connectivity, anisotropy, and complexity can influence root elongation, branching, and the formation of root surface area and volume [27,28]; on the other hand, roots introduce fresh carbon sources into the soil matrix via rhizodeposition, root exudates, and root residues, and may subsequently modulate microbial activities and labile organic carbon turnover [10,26,29]. Consequently, roots act as both active responders to soil structural changes and key participants in the formation and transformation of labile carbon.

Few long-term field studies have simultaneously linked aggregate-size distribution, CT-resolved pore architecture, crop root morphology, and aggregate-associated labile carbon within the same experimental platform. Using a field micro-plot experiment established in 2009, we compared four organic amendment systems under equivalent N, P, and K inputs. We addressed three questions: (1) Do contrasting amendment systems generate distinct aggregate–pore architectures? (2) Are peanut root traits more closely associated with pore geometric heterogeneity than with total porosity alone? (3) Do these structural and root responses correspond to contrasting allocations of EOC, MBC, and DOC between bulk soil and aggregate-size fractions? We hypothesized that traditional organic materials would favor larger aggregate-sized units or pore connectivity, whereas carbonized amendments would increase fine-scale pore heterogeneity, with corresponding differences in peanut root morphology and labile carbon allocation. Because all variables were measured at a single post-treatment sampling event, the proposed pore, aggregate, root and carbon feedback is treated as a conceptual framework that requires further temporal and mechanistic validation, rather than as a validated causal sequence.

## 2. Materials and Methods

### 2.1. Experimental Description

The field microplot experiment was established in 2009 at the Long-Term Fertilization Experimental Station of Shenyang Agricultural University (40°48′ N, 123°33′ E), which is characterized by a typical temperate monsoon climate. The mean annual temperature ranges from 7.0 to 8.1 °C, and the mean annual precipitation ranges from 574 to 684 mm. The experimental soil is classified as a sandy clay loam, with initial sand, silt, and clay (<2 μm) contents of 510, 280, and 210 g kg^−1^, respectively.

### 2.2. Experiment Design and Sample Collection

**Figure 1:**
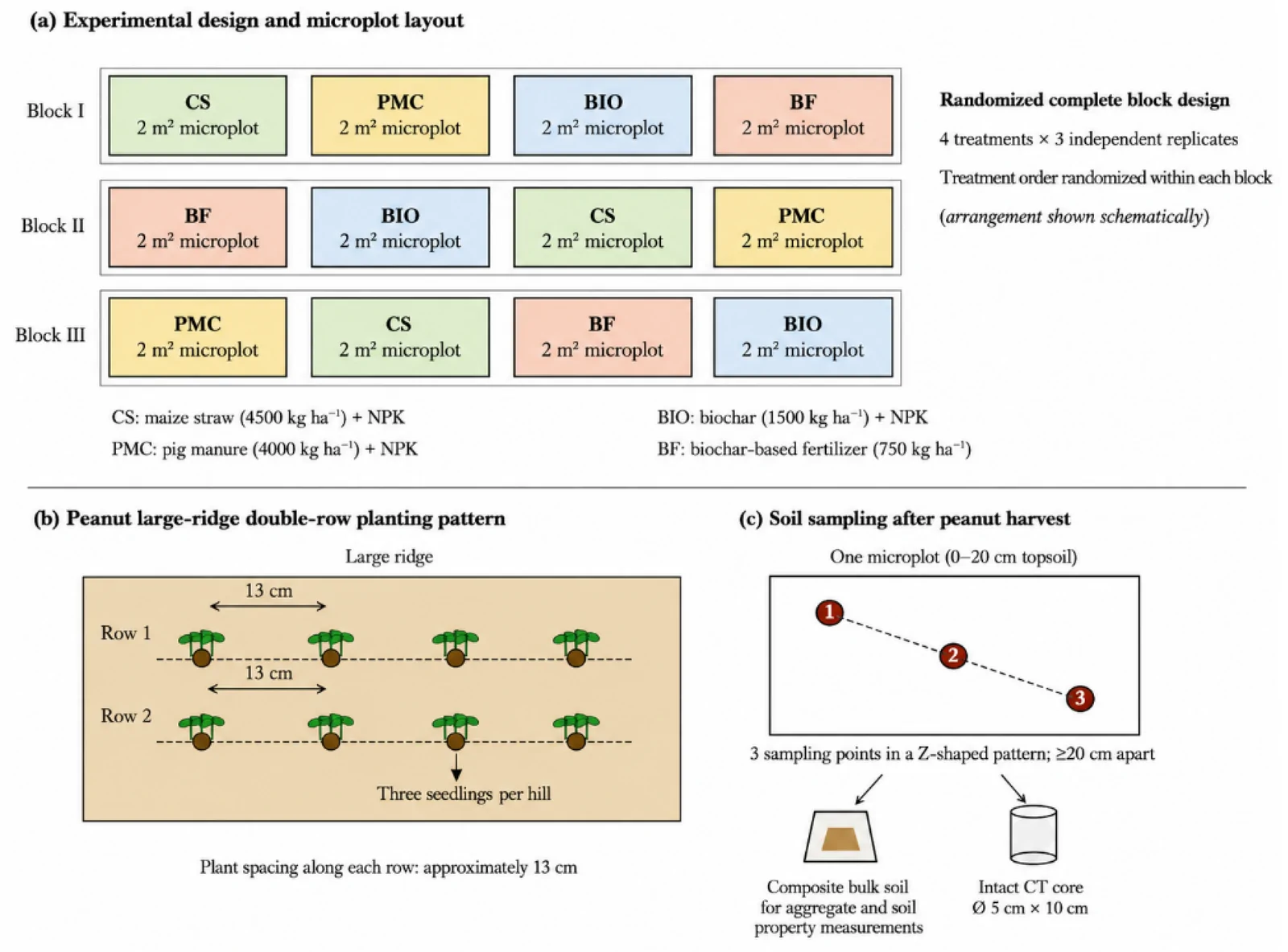
(**a**) Schematic diagram of the field experimental design, peanut planting pattern, and soil sampling scheme. Different colors indicate different amendment treatments. Arrows in panel (**b**) indicate the within-row plant spacing and the position of three seedlings per hill. Numbered circles in panel (**c**) indicate the three soil sampling points, and arrows indicate the subsequent use of samples for composite bulk-soil analysis and intact CT-core analysis.

The tested peanut cultivar was “Fuhua 12 (150GY)”, planted using a large-ridge double-row cultivation mode with a plant spacing of approximately 13 cm and three seedlings per hill (Figure 1). The experiment comprised four distinct treatments: (i) maize straw return at 4500 kg ha^−1^ + NPK (CS); (ii) pig manure at 4000 kg ha^−1^ + NPK (PMC); (iii) biochar at 1500 kg ha^−1^ + NPK (BIO); and (iv) biochar-based fertilizer at 750 kg ha^−1^ (BF). Each treatment was replicated three times. The experiment followed a randomized complete block design with three independent 2 m^2^ microplots per treatment. The basic physicochemical properties of the 0–20 cm soil layer before the experiment were as follows: The soil organic matter content was 13.3 g kg^−1^. The total nitrogen content was 0.86 g kg^−1^. The alkaline hydrolyzable nitrogen content was 51.3 mg kg^−1^. The total phosphorus content was 0.51 g kg^−1^. The available phosphorus content was 4.6 mg kg^−1^. The available potassium content was 114.5 mg kg^−1^. The soil pH was 6.1.

Biochar preparation: Biochar was produced via the pyrolysis of corncobs as the primary raw material at 450 °C, passed through an 80–100 mesh sieve, and subsequently granulated. The biochar-based fertilizer (10-13-13) was manufactured using biochar as the matrix, supplemented with nitrogen (N), phosphorus (P), and potassium (K) nutrients blended via chemical or physical methods. The chemical fertilizers applied in the experiment included urea (N, 46%), single superphosphate (P_2_O_5_, 16%), and potassium sulfate (K_2_O, 50%). The application rates of N, P, and K were 75, 42.6, and 80.9 kg ha^−1^, respectively. The total nutrient inputs of N, P, and K were equalized across all treatments based on the nutrient content of the BF treatment; specifically, the amount of supplemental chemical fertilizer was calculated by deducting the N, P, and K amounts contained in the organic materials from the total target nutrient requirement (Table 1). The application rates of maize straw and pig manure were calculated on a dry weight basis. Prior to sowing each year, all organic amendments and chemical fertilizers were uniformly incorporated into the 0–20 cm soil layer, thoroughly mixed, and then ridged for planting. Based on the application rates and total C concentrations in Table 1, the nominal annual organic-material C inputs were 1998, 1168, 500, and 58 kg C ha^−1^ yr^−1^ for CS, PMC, BIO, and BF, respectively. These values quantify the amount of C applied but do not represent C decomposability, microbial availability, or the proportion ultimately retained in soil. Therefore, the treatments should be interpreted as complete fertilization systems that differed in C quantity and quality, C/N ratio, application rate, and nutrient-release pattern, rather than as isolated effects of amendment type alone.

### 2.3. Soil Sampling and Analysis

Following the peanut harvest in 2023, soil samples were collected from the 0–20 cm topsoil layer. Within each micro-plot, three sampling points were randomly selected in a zigzag (Z-shaped) pattern with a minimum distance of 20 cm between adjacent points. The samples collected from the same micro-plot were thoroughly mixed to form a single composite sample representing one biological replicate; thus, the micro-plot served as the basic experimental unit for statistical analysis (n = 3). A portion of the soil was collected as intact soil cores using rigid PVC cylinders (5 cm in diameter and 10 cm in height). These cores were tightly sealed with plastic wrap, placed horizontally in aluminum boxes, and stored in a refrigerator at 4 °C prior to X-ray CT scanning analysis. Additional bulk soil samples were collected simultaneously for aggregate fractionation and the determination of soil physicochemical properties and labile organic carbon fractions.

Before CT scanning, all intact soil cores were adjusted to a comparable moisture status. Briefly, the cores were slowly equilibrated to a matric potential of −10 kPa using a sand table or ceramic plate apparatus until the change in core mass was less than 0.1% within 24 h. After equilibration, the cores were immediately sealed with plastic film to minimize moisture loss and scanned within the same day. Core mass was recorded before and after scanning, and gravimetric water content was determined after scanning to verify the consistency of moisture conditions among treatments. Therefore, the CT-derived pore parameters represent air-filled macropores distinguishable under the standardized −10 kPa scan condition, rather than the total soil pore system.

### 2.4. Soil Physicochemical Properties and Labile Carbon Fractions

Soil bulk density was determined using the core method. Soil pH was measured potentiometrically using a pH meter (PHSJ-3F, Shanghai INESA Scientific Instrument Co., Ltd., Shanghai, China) in a soil-to-water suspension at a ratio of 1:2.5 (*w*/*v*) after equilibration. Soil organic matter was determined using the potassium dichromate oxidation method, and total nitrogen was determined using the Kjeldahl method. Alkaline hydrolyzable nitrogen was measured using the alkaline hydrolysis diffusion method. Total phosphorus was determined by the molybdenum blue colorimetric method following acid digestion. Plant-available phosphorus was extracted with 0.5 mol L^−1^ NaHCO_3_ using the Olsen method and determined colorimetrically. Plant-available potassium was extracted with 1 mol L^−1^ neutral NH_4_OAc and measured by flame photometry. Microbial biomass carbon (MBC) was determined using the chloroform fumigation–extraction method with K_2_SO_4_ as the extractant. Dissolved organic carbon (DOC) was extracted with 0.5 mol L^−1^ K_2_SO_4_ and quantified using an automated TOC/TN analyzer. Easily oxidizable organic carbon (EOC) was determined using the 333 mmol L^−1^ KMnO_4_ oxidation method.

### 2.5. Soil Aggregate Fractionation and Analysis

Soil aggregate fractionation was performed using a dry sieving method. After the removal of visible debris and plant roots, fresh soil samples were air-dried at 4 °C to a moisture content of 100 g kg^−1^ (monitored via the gravimetric method), thoroughly homogenized, and passed through a 5 mm sieve. A 100 g aliquot of the sieved soil was placed onto the top of a sieve nest (mesh sizes of 5 mm, 2 mm, and 0.25 mm) and fractionated using a dry sieving shaker (Retsch AS200, Retsch GmbH, Haan, Germany). The operating parameters were set to an amplitude of 1.5 cm, a sieving time of 1 min, and a frequency of 30 cycles min^−1^. This procedure yielded three distinct aggregate size fractions: large macroaggregates (LMA, >2 mm), small macroaggregates (SMA, 0.25–2 mm), and microaggregates (MA, <0.25 mm). This process was repeated until a sufficient sample mass was accumulated for each fraction. A portion of the obtained aggregates from each fraction was immediately used for the analysis of labile organic carbon components.

### 2.6. Soil Pore Structure Analysis

All intact soil cores were scanned using a high-resolution industrial X-ray CT scanner (inspeXio SMX-225CT FPD HR, Shimadzu Corporation, Kyoto, Japan). Each soil core was secured on a rotation stage positioned between the X-ray tube and the detector. The focus-to-object distance (FOD) and focus-to-detector distance (FDD) were set to 150.8 mm and 800 mm, respectively. The magnification factor, determined by the ratio of FDD to FOD, was 5.3×, corresponding to a voxel resolution of 27 μm. Due to inherent image noise, the effective spatial resolution was approximately 30 μm. Therefore, all pore metrics reported here refer only to the CT-resolved > 30 μm domain and do not represent smaller pores that may contribute to microbial habitat, water retention, nutrient diffusion, and carbon protection. To optimize the signal-to-noise ratio (SNR) of the CT images, the X-ray tube voltage and current were maintained at 160 kV and 70 μA, respectively. A 0.5 mm copper (Cu) filter was deployed during scanning to minimize beam hardening artifacts. The soil cores were rotated from 0° to 360° at a constant step size of 0.15°. Projection images of the samples were recorded using a Varian 16-inch digital detector array (Varian Medical Systems, Palo Alto, CA, USA) with a resolution of approximately 14 million pixels. The exposure time for each projection was 0.5 s, yielding a total of 2400 projection images (2048 × 2048 pixels) for 3D reconstruction. Tomographic images were reconstructed using a filtered back-projection (FBP) algorithm via VGSTUDIO MAX 3.4 software (Volume Graphics GmbH, Heidelberg, Germany). The final reconstructed slices were converted into 16-bit raw format for subsequent analysis.

Image preprocessing—including cropping, normalization, noise reduction, and edge enhancement—was performed using ImageJ 1.51 software (National Institutes of Health, Bethesda, MD, USA). Normalization of the CT datasets was conducted based on a stacked histogram, with the saturated pixel parameter set to 0.3%. Noise reduction was achieved using a 3D median filter with a radius of 1 pixel. An unsharp mask filter (settings: radius, σ = 1.1; weight, w = 0.6) was applied to mitigate partial volume effects. To eliminate potential structural disturbances, a 1 cm layer was trimmed from both the top and bottom ends of each soil core. Additionally, the soil layer from −6 to −8 cm was excluded to avoid artificial macropores potentially induced during field sampling. The visualization and quantification of macropore structures were conducted exclusively within these selected volumes of interest (VOIs).

**Figure 2:**
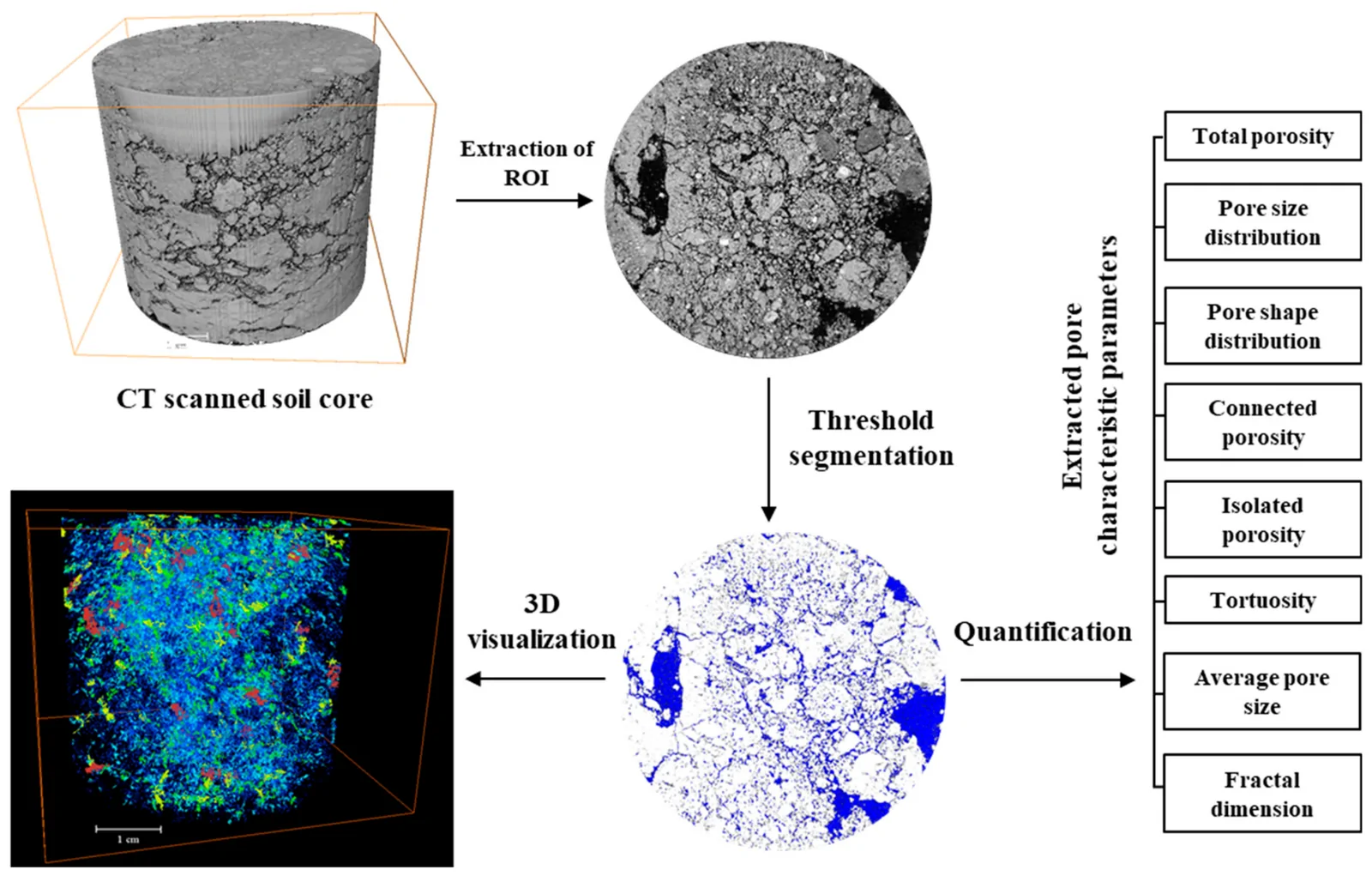
Steps of CT image processing and pore quantification. ROI denotes the region of interest extracted from each reconstructed CT image stack for subsequent pore segmentation and quantification.

Quantification of the macropore network was performed using Avizo 3D software (version 2020.1, Thermo Fisher Scientific, Waltham, MA, USA) after extracting the region of interest (ROI) from each reconstructed CT image stack (Figure 2). Macropores within the soil cores were categorized into two types: connected pores and isolated pores. Connected pores were defined as macropores interconnected with at least one adjacent pore, whereas isolated pores referred to those completely disconnected from any neighboring pore spaces. Extraction of the connected pore network was executed using the “Axis Connectivity” module in Avizo, which isolated the continuous pore network by filtering out all disconnected macropores. To evaluate individual pore characteristics within the highly interconnected network, the “Separate” module was employed to segment individual pores. This separation process was based on a watershed algorithm, which identified pore throats to partition the interconnected system. The 3D geometric attributes of the connected pores, including pore size, volume, perimeter, and surface area, were subsequently quantified using the “Label Analysis” module. This image-based pore classification is resolution dependent and should not be interpreted as evidence that isolated pores were hydraulically disconnected at finer scales or under different moisture conditions.

To visualize the identifiable macropore system (>30 μm), an equivalent diameter value was assigned to each discrete pore (including both isolated pores and separated connected pores), meaning the voxel value of each individual pore in the processed CT image directly corresponded to its equivalent diameter. Consequently, pores of varying sizes were rendered in distinct colors, where cooler colors represented smaller pore structures and warmer colors indicated larger ones. By filtering the corresponding pore sizes, 3D pore structures of different size classes were generated and visualized using the “Volume Rendering” module in Avizo. This filtering process utilized the manual thresholding tool in Avizo to input specific pore size ranges. To investigate the size effects of the soil pore network, macropores were segmented and visualized into nine distinct diameter classes: 30–50, 50–100, 100–200, 200–300, 300–500, 500–700, 700–1000, 1000–1500, and >1500 μm. To examine the spatial distribution and vertical variations in these different pore size classes within the soil cores, the volumetric percentage of the selected pores in each horizontal cross-sectional slice was analyzed using the “Volume Fraction” module in Avizo. In this study, “CT-resolved macropore heterogeneity” refers specifically to variation in isolated porosity, anisotropy, mean pore diameter, and pore fractal dimension within the >30 μm pore domain.

### 2.7. Peanut Root Morphological Characteristics

At the peanut maturity stage, the intact root systems of three representative plants from each micro-plot were collected by excavating a soil block measuring 30 × 30 × 40 cm (length × width × depth). The bulk soil loosely adhering to the peanut roots was gently shaken off, and the root systems were placed into nylon mesh bags and carefully washed with water to remove any remaining soil particles. The cleaned root systems were then transported to the laboratory for morphological analysis. The roots were scanned using an Epson Perfection V700 scanner (Seiko Epson Corporation, Suwa, Japan), and the acquired images were subsequently analyzed using the WinRHIZO-Tron 2016 software (Regent Instruments Inc., Quebec City, QC, Canada) to quantify the total root length, root surface area, root volume, and the number of root forks. Root volume, root length, root branch number, and root surface area were expressed on a per-plant basis as cm^3^ plant^−1^, m plant^−1^, number plant^−1^, and cm^2^ plant^−1^, respectively.

### 2.8. Calculations of Dry-Aggregate Indices

(1) Mean weight diameter of soil aggregates (MWD):

$$MWD=\sum_{i=1}^{n}X_{i}W_{i}$$

(2) Geometric Mean Diameter of soil aggregates (GMD):

$$GMD=exp\left(\sum_{i=1}^{n}W_{i}lnX_{i}\right)$$

where $X_{i}$ is the mean diameter of the *i*-th aggregate size fraction (mm), and $W_{i}$ represents the percentage content of the *i*-th aggregate size fraction (%);

(3) Fractal dimension of soil aggregates (D):

$$\left(3-D\right)=lg\left(\frac{X_{i}}{X_{max}}\right)=lg\left\{\frac{W_{\left(i\leq X_{i}\right)}}{W_{0}}\right\}$$

where $X_{max}$ is the mean diameter of the largest aggregate size fraction (mm); $W_{\left(i\leq X_{i}\right)}$ is the cumulative mass of aggregates with a diameter smaller than or equal to $X_{i}$ (g); and $W_{0}$ represents the total mass of aggregates across all size fractions (g).

### 2.9. Data Analysis

In this study, the effects of long-term organic amendment treatments on soil physicochemical properties, aggregate-size distribution and stability, CT-derived pore characteristics, crop root traits, and labile carbon fractions were evaluated by one-way analysis of variance (ANOVA). When ANOVA indicated a significant treatment effect, means were compared using Duncan’s multiple range test at *p* < 0.05. These analyses were performed with IBM SPSS Statistics 20. Pearson correlation analysis examined associations among variables. Before ANOVA, residual distributions and variance patterns were inspected graphically. Because each treatment included only three independent replicates, formal tests of normality and homogeneity of variance had limited diagnostic power; therefore, ANOVA and correlation results were interpreted cautiously. For the exploratory structural equation model (SEM), candidate variables were assigned a priori to five conceptual domains: soil physicochemical properties, aggregate structure, CT-resolved macropore characteristics, root traits, and labile carbon. This classification was based on the study objectives, biological relevance, and the conceptual framework of this study. No stepwise or other data-driven variable selection was applied. Given the small sample size (12 independent microplots), formal VIF-based screening and alternative-model selection were not undertaken because their results would be unstable. Instead, a parsimonious path structure was specified a priori. Standardized path coefficients were interpreted as hypothesis-generating associations rather than confirmatory causal effects. Figures were prepared using Origin 2021 (OriginLab Corporation, Northampton, MA, USA) and R version 4.5.0 (R Foundation for Statistical Computing, Vienna, Austria).

## 3. Results

### 3.1. Differential Responses of Soil Aggregates and Pore Structures

#### 3.1.1. Soil Aggregate Composition and Stability Characteristics

**Figure 3:**
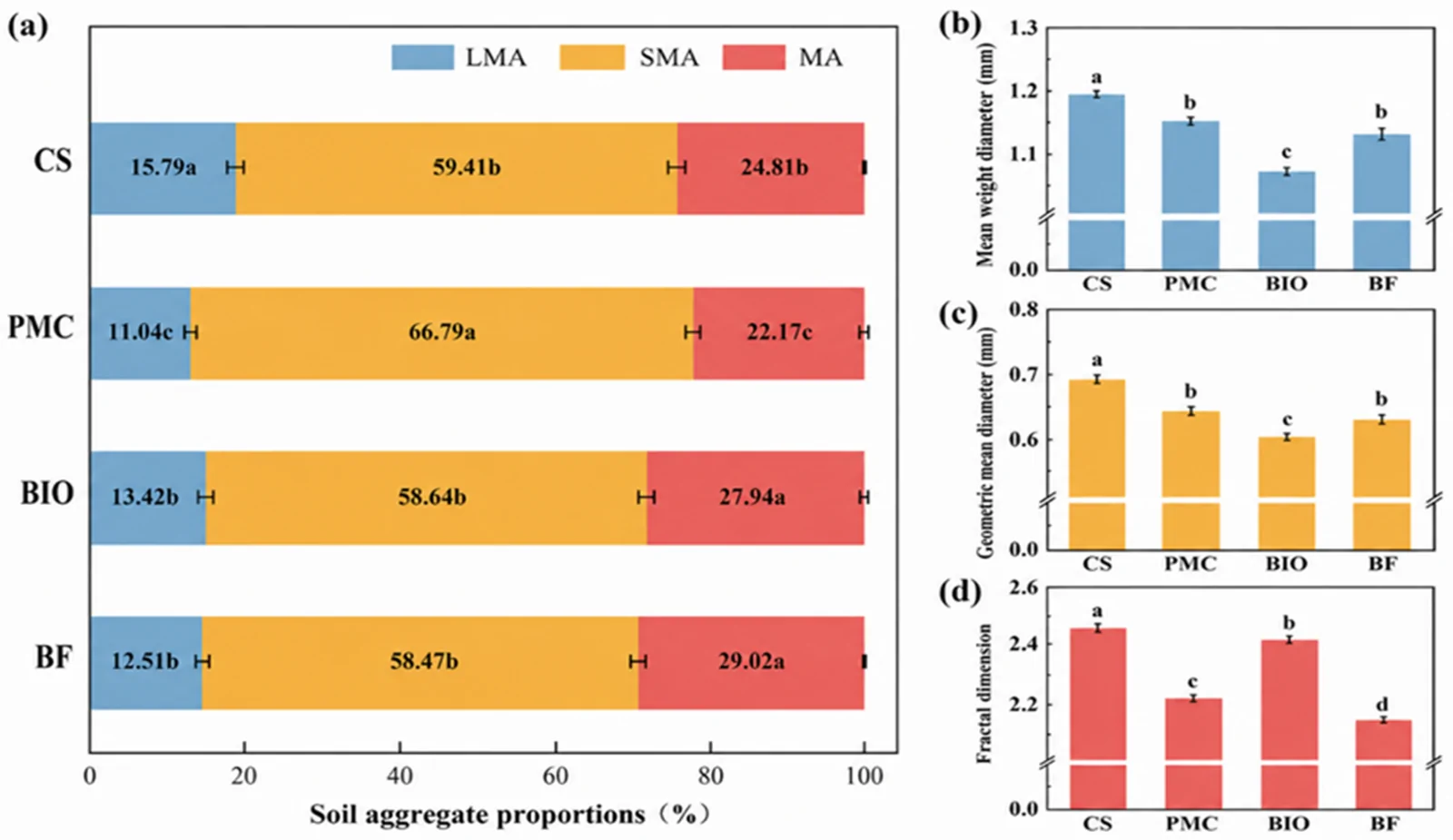
Composition and stability characteristics of soil aggregates. (**a**) Proportions of large macroaggregates (LMA, >2 mm), small macroaggregates (SMA, 0.25–2 mm), and microaggregates (MA, <0.25 mm); (**b**) mean weight diameter (MWD); (**c**) geometric mean diameter (GMD); and (**d**) fractal dimension of soil aggregates under different treatments. CS, PMC, BIO, and BF represent corn straw, pig manure compost, biochar, and biochar-based fertilizer treatments, respectively. Different lowercase letters indicate significant differences among treatments for the same variable at *p* < 0.05 (n = 3).

Long-term PMC application was associated with a higher proportion of SMA, showing a 12.4% increase compared with the CS treatment. In contrast, the CS treatment had a significantly higher proportion of LMA, which was 43.0% higher than that under the PMC treatment. The BIO and BF treatments increased the proportion of the MA aggregate fraction, with the BF treatment exhibiting a 30.9% increase compared with the PMC treatment (Figure 3a). Aggregate stability indices further revealed that the CS treatment had the highest soil aggregate stability, with its MWD and GMD reaching 1.23 mm and 0.69 mm, respectively, both of which were higher than those under the other treatments (Figure 3b,c); the aggregate stability under the PMC treatment was second only to that under the CS treatment. Conversely, the BIO treatment exhibited the lowest MWD and GMD (1.08 mm and 0.63 mm, respectively), while the BF treatment had the lowest fractal dimension (Figure 3d).

#### 3.1.2. CT-Resolved Pore Architecture and Pore-Size Distribution

**Figure 4:**
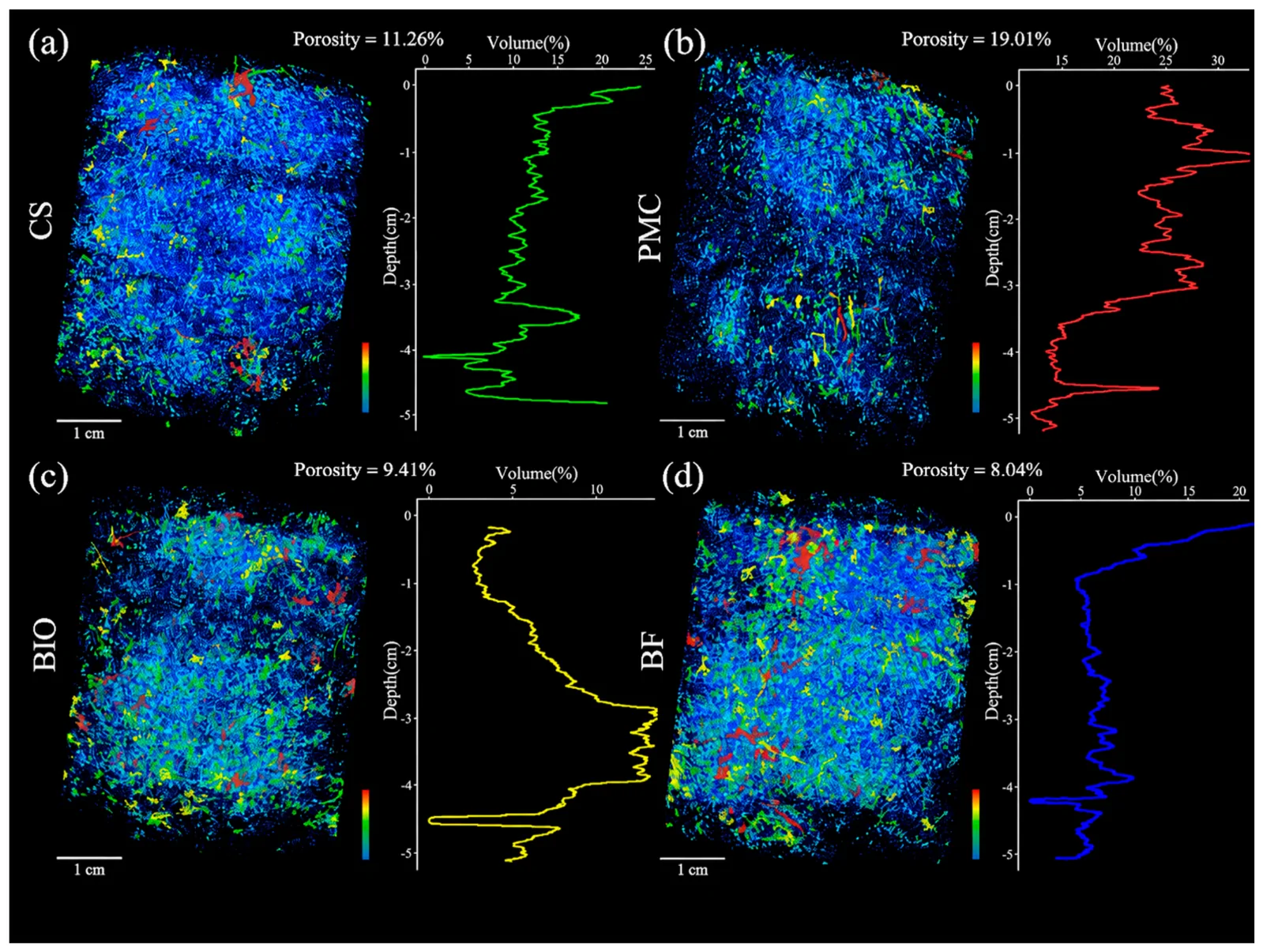
Soil macropore systems based on CT scanning under different treatments. (**a**) CS; (**b**) PMC; (**c**) BIO; and (**d**) BF. CS, PMC, BIO, and BF represent corn straw, pig manure compost, biochar, and biochar-based fertilizer treatments, respectively. Warmer colors indicate larger pore sizes, whereas cooler colors indicate smaller pore sizes. The right side of each 3D pore structure diagram shows the slice-wise CT-resolved porosity along the soil-core depth.

The CT scanning results indicated that the CT-resolved total porosity and connected porosity reached their highest values under the long-term application of the PMC treatment, reaching 19.01% and 10.86%, respectively (Figure 4b; Table S2). In contrast, the BIO and BF treatments exhibited significantly lower CT-resolved porosities of 9.41% and 8.04%, respectively, with the porosity across most of the soil profile depths remaining below 10% (Figure 4c,d). However, their isolated porosity, degree of anisotropy, mean pore size, and fractal dimension were significantly higher than those under traditional organic material amendment treatments (Table 2).

**Figure 5:**
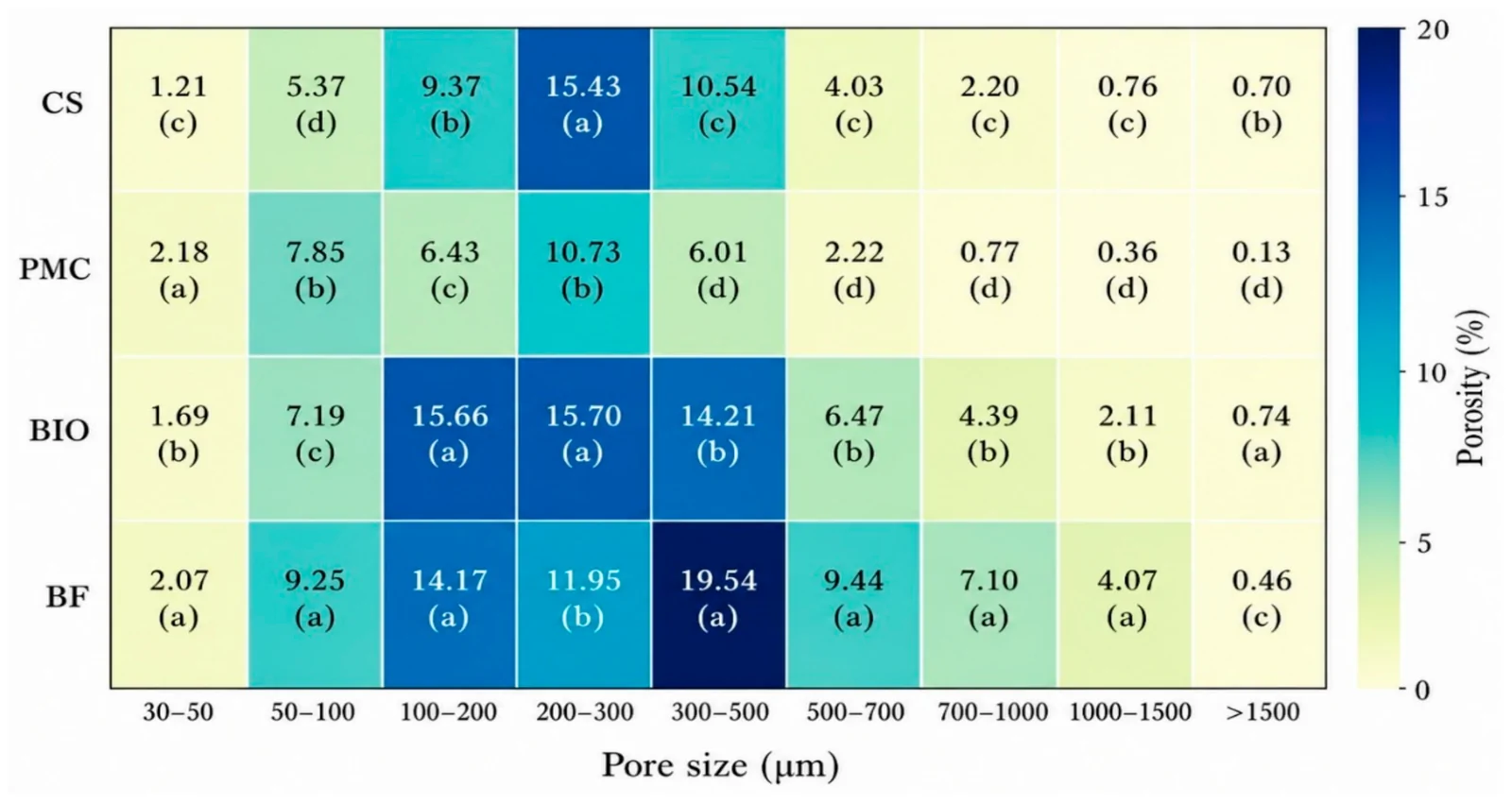
Pore size distribution of soil structure for different treatments. CS, PMC, BIO, and BF represent corn straw, pig manure compost, biochar, and biochar-based fertilizer treatments, respectively. Different lowercase letters indicate significant differences among treatments for the same variable at *p* < 0.05 (n = 3).

Long-term application of BIO showed higher porosity within the 100–300 μm pore diameter range (Figure 5), whereas the BF treatment showed higher porosity within the ranges of 30–200 μm and 300–1500 μm. Conversely, the PMC treatment had the highest porosity in the 30–50 μm class (30–50 μm). Under the CS treatment, the soil porosity peaked within the 200–300 μm range, while variations across the remaining pore size classes were relatively limited.

### 3.2. Selective Response of Peanut Root Morphological Characteristics to Changes in Pore—Aggregate Structure

**Figure 6:**
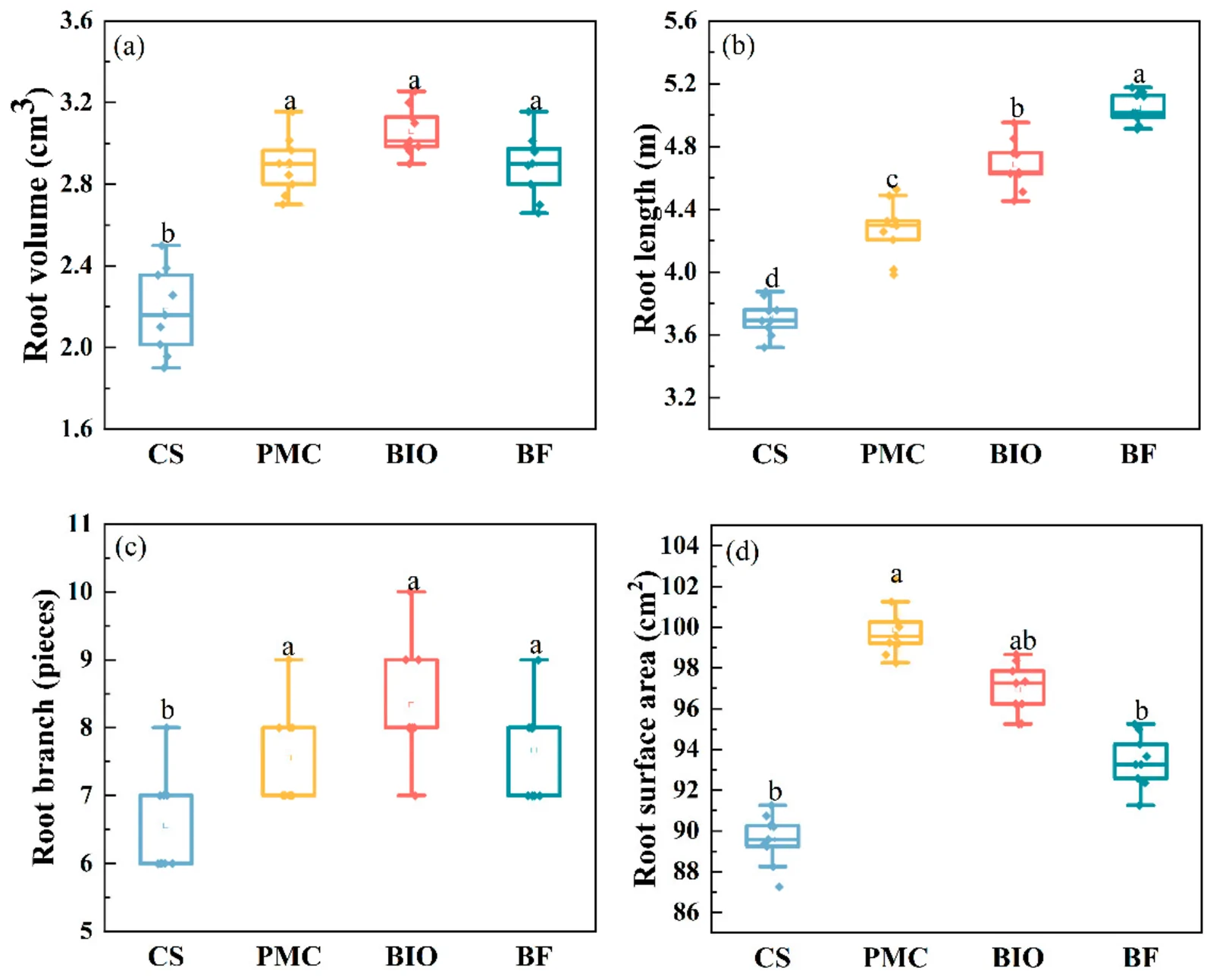
Peanut root morphological traits at maturity. Panels (**a**–**d**) show root volume (cm^3^ plant^−1^), root length (m plant^−1^), root branch number (number plant^−1^), and root surface area (cm^2^ plant^−1^), respectively. CS, PMC, BIO, and BF represent corn straw, pig manure compost, biochar, and biochar-based fertilizer treatments, respectively. Different lowercase letters indicate significant differences among treatments for the same variable at *p* < 0.05 (n = 3).

Long-term application of the BIO treatment had significantly greater peanut root volume and more root forks, whereas the BF treatment had greater root length, which was significantly higher than that under the other treatments. The PMC treatment had the largest peanut root surface area, reaching approximately 102 cm^2^. In contrast, the long-term application of CS generally had lower values for the measured peanut root traits (Figure 6). Correlation analysis revealed that peanut root volume was significantly and positively correlated only with the degree of pore anisotropy (Table 3; *p* < 0.05). Root length was significantly and negatively correlated with total porosity and connected porosity (*p* < 0.05), but exhibited highly significant positive correlations with all the remaining pore structural parameters (*p* < 0.01). No significant correlations were observed between the number of root forks or root surface area and any of the evaluated soil pore parameters. Given the limited sample size and the simultaneous measurement of root and pore variables, these correlations do not demonstrate a direct causal relationship between pore architecture and root development.

### 3.3. Characteristics and Spatial Distribution of Labile Organic Carbon Components in Soil

#### 3.3.1. Soil Labile Organic Carbon Components and Their Carbon Pool Mass

Long-term BF application primarily increased EOC content and the percentage of EOC in TOC (Table 4), which were 38.5% and 47.4% higher, respectively, than those under PMC. No significant differences in EOC content were observed among the other treatments. The MBC content under CS reached 485.65 mg kg^−1^, which was significantly higher than that under the other treatments and 94.8% higher than that under BF. The percentage of MBC in TOC showed a similar response pattern. DOC content did not differ significantly among treatments.

#### 3.3.2. Labile Organic Carbon Fractions and Carbon Pool Quality in Different Aggregate-Size Fractions

**Figure 7:**
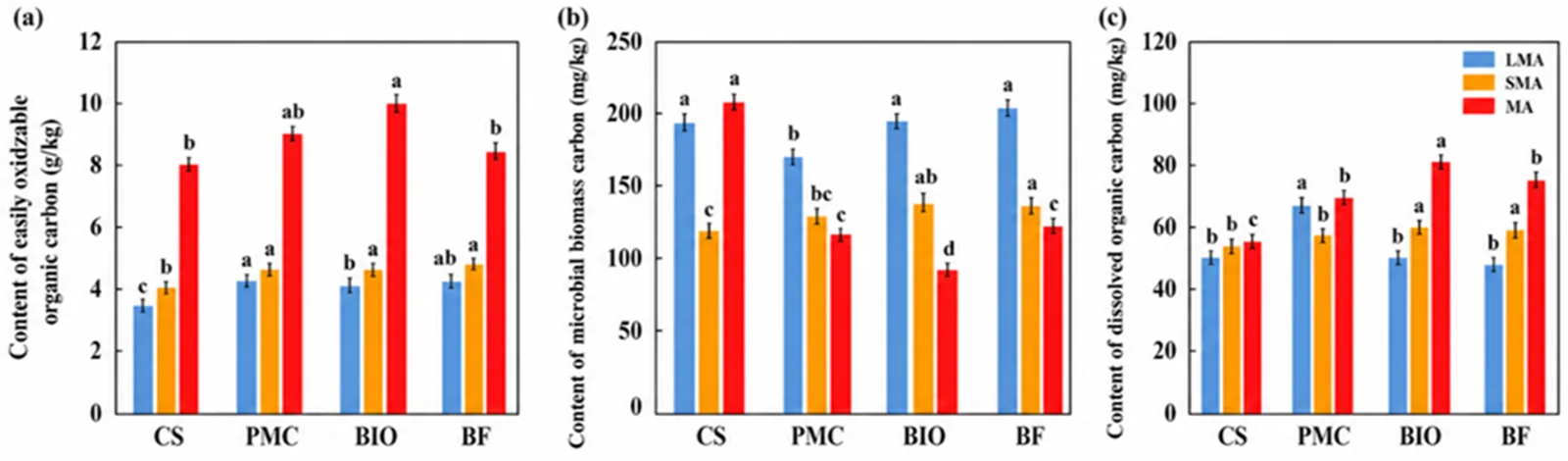
Labile organic carbon fractions and carbon pool quality in different aggregate size fractions. (**a**–**c**) represent the contents of EOC, MBC, and DOC in aggregates of different particle sizes under different treatments; different lowercase letters indicate significant differences between treatments within the same particle size (*p* < 0.05).

Long-term application of different organic amendments was associated with significant differences in the distribution of labile organic carbon fractions among aggregate-size classes (Figure 7a–c). BIO had higher EOC contents in the SMA and MA fractions. The highest EOC content occurred in the MA fraction under BIO and approached 10 g kg^−1^. PMC had a relatively high EOC content in the LMA fraction, whereas BF generally showed intermediate values. EOC and DOC contents were relatively low across all aggregate-size fractions under CS (Figure 7a,c).

MBC content varied markedly among treatments and aggregate-size fractions. CS had significantly higher MBC content in the MA fraction, reaching 211 mg kg^−1^. In contrast, BIO had the lowest MBC content in the MA fraction, at 95 mg kg^−1^. In the SMA fraction, MBC contents under BIO and BF were 133 and 136 mg kg^−1^, respectively. These values were higher than those under the conventional organic amendment treatments. In the LMA fraction, PMC showed the lowest MBC content (Figure 7b).

DOC content was highest in the MA fraction under BIO, reaching 91 mg kg^−1^, and lowest in the same fraction under CS. In the SMA fraction, DOC contents under BIO, BF, and PMC were significantly higher than that under CS. In the LMA fraction, PMC had the highest DOC content (Figure 7c). Overall, BIO was characterized by higher EOC and DOC in the SMA and MA fractions, PMC by higher EOC and DOC in the LMA fraction, and CS by higher MBC in the LMA and MA fractions. These concentrations represent standing pools at one sampling time and cannot distinguish carbon availability, production, sorption, microbial consumption, mineralization, or turnover rates.

BF maintained relatively high percentages of EOC in TOC across all aggregate-size fractions (Table S3). The percentage of EOC in TOC was significantly highest in the LMA fraction and was 26.5% higher than that under CS. BF also showed relatively high percentages of MBC in TOC in the LMA and SMA fractions, reaching 2.20% and 1.43%, respectively. These values were 13.4% higher than those under CS in the LMA fraction and 16.3% higher than those under PMC in the SMA fraction.

CS showed the lowest percentage of EOC in TOC across all aggregate-size fractions. However, the percentage of MBC in TOC in the MA fraction was the highest, reaching 2.32%. This value was 134.3% higher than that under BIO. BIO produced the highest percentages of DOC in TOC in the MA and SMA fractions. In the MA fraction, the percentage of DOC in TOC under BIO was 41.2% higher than that under CS. PMC showed the highest percentage of DOC in TOC in the LMA fraction, at 0.61%.

### 3.4. Exploratory Associations Among Soil Properties, Structure, Root Traits, and Labile Carbon

Long-term application of corn straw (CS) resulted in the highest bulk density, while PMC and BF treatments had the lowest bulk density; BIO and BF treatments had higher soil pH, whereas PMC treatment had the lowest pH. SOC content was higher in PMC and CS treatments, and lower in BIO and BF treatments. Overall, PMC treatment showed a more pronounced effect on soil nutrient enhancement, reflected by higher TN and AK, while the nutrient content in BIO and BF treatments was generally lower than in PMC and CS treatments (Table S1). These covarying physicochemical changes provide important context for interpreting treatment-associated variation in aggregate structure, CT-resolved pores, root traits, and labile carbon allocation.

**Figure 8:**
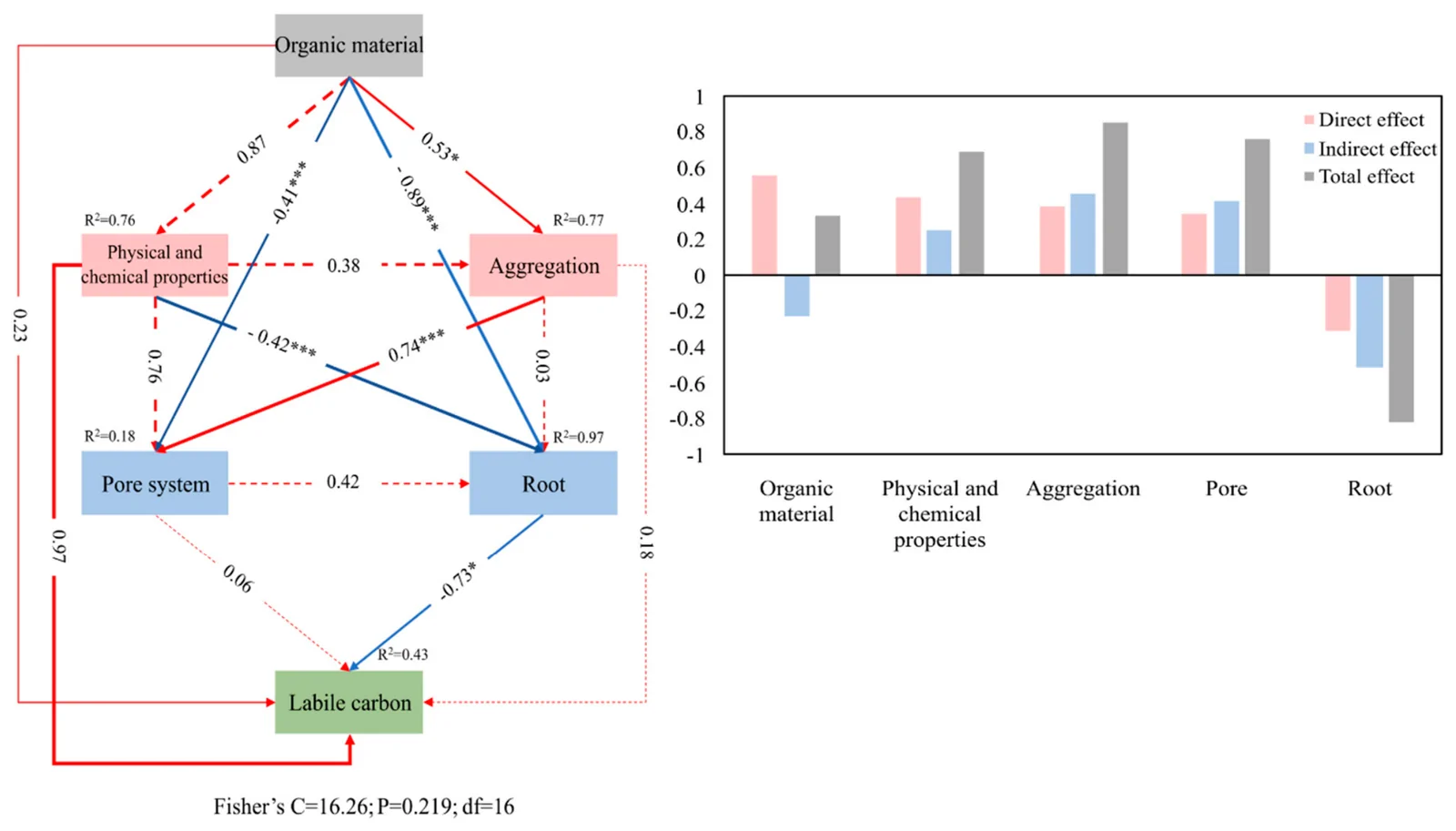
Structural equation model (SEM) illustrating the direct and indirect associations among organic amendment systems, soil physicochemical properties, aggregate characteristics, pore architecture, peanut root traits, and labile organic carbon. Numbers adjacent to the arrows represent standardized path coefficients. Red and blue arrows indicate positive and negative associations, respectively, whereas solid and dashed arrows indicate significant and nonsignificant paths, respectively. Asterisks indicate significance levels: * *p* < 0.05, and *** *p* < 0.001. The coefficients of determination (R^2^) shown beside the response variables represent the proportion of variance explained by the model. The bar chart on the right shows the standardized direct, indirect, and total effects of each variable group on labile organic carbon. Model fit statistics were Fisher’s C = 16.26, *p* = 0.219, and df = 16. Because the model was based on only 12 independent microplots, the pathways were interpreted as exploratory associations rather than definitive causal relationships. Arrow directions reflect the proposed conceptual framework and should not be interpreted as validated temporal or causal directions.

SEM results (Figure 8) showed positive standardized associations between the amendment-system composite and both soil physicochemical properties and aggregate structure, with path coefficients of 0.87 and 0.53, respectively. Soil physicochemical properties and aggregate structure were positively associated with the pore-system composite (0.76 and 0.74, respectively). The direct association between the pore system and labile organic carbon was weak (0.06), whereas the root-trait composite was negatively associated with labile organic carbon (−0.73). Because the model was based on only 12 independent microplots and all variables were measured at one sampling event, these coefficients describe exploratory covariance patterns and do not establish temporal order or causal effects.

## 4. Discussion

### 4.1. Long-Term Straw Return Affects Large-Aggregate Formation and Microbial Biomass Carbon Allocation

Under equivalent NPK inputs, long-term application of corn straw (CS) had the highest large macroaggregates (LMA) proportion, mean weight diameter (MWD) and geometric mean diameter (GMD). However, CS also received the greatest nominal annual organic-material C input and differed from the other systems in C/N ratio, residue structure, and nutrient-release pattern. Therefore, the observed aggregate pattern should be interpreted as a response to the complete CS fertilization system, rather than to straw addition alone. Straw residues and their decomposition products may provide particulate nuclei and microbial binding agents, which is consistent with the observed formation of larger aggregate units [12,30].

It is worth noting that although CS treatment achieved the highest aggregate stability, it did not exhibit the highest CT-identifiable total porosity and communicating porosity. This indicates that the stability of aggregates and the number of large pores are not the same structural property. The aggregate components obtained from dry sieve mainly reflect the particle size composition of soil solid structural units, while CT analysis reflects spatial morphology and connectivity patterns of pores larger than 30 μm. Stable macroaggregates may contain many micropores, closed pores, or short-distance channels below CT recognition scales, so increasing the proportion of macroaggregates does not necessarily synchronize the formation of continuous large-pore networks running through soil columns [31,32,33,34,35]. Moreover, whether organic carbon inside large aggregates is protected depends not only on aggregate particle size but also on pores and spatial accessibility between organic matter and microorganisms [36,37]. Because pores smaller than 30 μm were not resolved, any inference concerning microbial habitat or carbon protection within these finer pore domains remains speculative.

CS treatment showed the highest bulk-soil MBC content and higher MBC and percentage of MBC in TOC at MA particle size, whereas the overall EOC and DOC contents at each aggregate-size fraction were relatively low. This pattern is consistent with, but does not demonstrate, rapid microbial assimilation of straw-derived substrates. Continuous input of cellulose, hemicellulose, and their decomposition products can provide energy for microorganisms, while the biomass and residues formed by microbial assimilation may further enter small-grain aggregates or bind to mineral surfaces [30,38]. Microbial biomass may subsequently contribute to microbial necromass and persistent SOC through further transformation and mineral association, although microbial necromass and turnover were not directly measured in this study [22,38]. Therefore, the lower EOC and DOC contents under CS should not be interpreted simply as insufficient carbon input. Instead, they may reflect the rapid utilization and microbial transformation of available substrates after straw decomposition.

Compared with labile carbon response, CS treatment had relatively weaker promoting effects on peanut root length, root volume, and root surface area, while MWD was negatively correlated with multiple root traits. This association may indicate a potential trade-off between aggregate stability and root spatial expansion. Although greater aggregate stability can help maintain soil structure, densely packed large aggregates with limited connected channels may reduce the availability of continuous pathways for root extension [6]. However, because soil penetration resistance, water availability, nutrient distribution, rhizosphere microbial activity, and root–pore spatial matching were not measured, this result should be interpreted as an exploratory association rather than evidence that stable aggregates directly inhibited root growth.

### 4.2. Long-Term Application of the Pig Manure Compost Amendment System Is Associated with Connected Macropores and SMA-Dominated Aggregation

PMC had the highest SMA proportion, CT-resolved total porosity (19.01%), and connected porosity (10.86%), with particularly high porosity in the 30–50 μm pore-size class. This structural pattern may be related to the combined characteristics of the PMC system, including its nominal annual C input (1168 kg C ha^−1^ yr^−1^), relatively low C/N ratio, nutrient availability, carbon chemistry, and nutrient-release behavior. Manure-derived organic inputs can provide readily available substrates for microbial growth and extracellular polymer production, thereby contributing to particle binding, aggregate reorganization, and the development of connected biopores [39]. Previous CT studies have also reported increased macropore abundance and connectivity after long-term manure application, which is consistent with the structural pattern observed here [40]. However, because the treatments differed in multiple amendment properties, the observed PMC-associated structure should be interpreted as the result of the complete amendment system rather than as an isolated manure-type effect. Overall, PMC appeared to favor SMA-dominated aggregation and a more connected CT-resolved macropore network, rather than the larger dry-sieved aggregate units observed under CS.

PMC-treated peanut root surface area was the largest, about 102 cm^2^, co-occurring with its high CT-resolved pore connectivity and 30–50 μm porosity. Connected macropores may facilitate the migration of water, oxygen, and soluble nutrients in the root zone, increase the probability of the root tip contacting suitable channels, and facilitate the expansion of fine and lateral roots in the soil [6]. Increasing root surface area further expands the contact range between peanut roots and soil, thereby improving the potential range of nutrient absorption interfaces and rhizosphere deposition [27]. However, in this study, there was no significant correlation between root surface area and individual pore indicators, indicating that the larger root surface area may reflect the combined effects of pore structure, soil nutrients, and lower bulk density, and cannot be fully attributed to a single pore size or interconnected pores.

In terms of labile carbon distribution, PMC treatment had higher EOC and DOC contents in LMA, while the total MBC was at a higher level but lower than CS treatment. Pig Manure can directly input easily decomposable organic matter and soluble organic components, and larger root surface areas may also expand the range of source carbon input. These carbon sources may reach the aggregate surface and interior through interconnected pores. Plant residues, root carbon, and microorganisms in large aggregates often form carbon conversion hotspots, which is consistent with the high EOC and DOC contents in LMA during PMC treatment [18,37]. However, higher connectivity may also enhance oxygen and substrate transport, and increase microbial accessibility to labile carbon, so its role is not simply to protect organic carbon. Research shows that when organic matter within aggregates is adjacent to external connecting pores, its decomposition rate may be higher than that of organic matter isolated by closed pores [40,41].

### 4.3. Long-Term Carbonized Amendment Systems Are Associated with CT-Resolved Macropore Heterogeneity and Labile Carbon Allocation

Compared with the long-term application of maize straw (CS) and pig manure (PMC), the long-term application of biochar (BIO) and biochar-based fertilizer (BF) did not significantly increase the CT-resolvable total porosity and connected porosity; however, they increased the isolated porosity, degree of anisotropy, mean pore size, and fractal dimension, while simultaneously elevating the proportion of microaggregates (MA). These results indicate that BIO and BF altered the CT-resolved > 30 μm pore domain mainly through pore-size redistribution and increased macropore heterogeneity, rather than through a uniform increase in total pore volume or macroaggregate formation. This pattern may be related to the porous and irregular geometry of biochar particles and the interfacial spaces formed between char particles and soil mineral grains, although these mechanisms were not directly measured. Biochar particles may fill, segment, or redirect pre-existing connected macropore pathways, thereby increasing the proportion of disconnected pore components at the CT resolution [42,43]. Meanwhile, internal pores within char particles and char–mineral interfaces may create localized domains for water storage and mass exchange. Therefore, the greater isolated porosity under BIO and BF should be interpreted as evidence of pore-size redistribution and enhanced CT-resolved macropore heterogeneity, rather than direct evidence for improved carbon protection, reduced transport, or enhanced microbial habitat [35,42].

Although the BIO and BF treatments exhibited similar trends in structural modulation, distinct variations were observed in their specific pore size distributions and root responses. The BIO treatment was characterized by higher porosity in the 100–300 μm classes, together with greater root volume and branching, whereas BF had higher proportions of 30–200 and 300–1500 μm pores and greater root length. Correlation analysis showed that root volume was positively correlated with anisotropy, while root length was positively correlated with isolated porosity, anisotropy, mean pore diameter, and fractal dimension. These results suggest that peanut root traits were more closely associated with pore directionality, size distribution, and spatial complexity than with total porosity alone. However, these cross-sectional relationships do not demonstrate that pore heterogeneity directly controlled root development [31,35,42]. Other factors, including penetration resistance, water availability, nutrient distribution, and rhizosphere microbial activity, may also contribute to root responses. Meanwhile, roots can reciprocally modify pore architecture through penetration, compaction, and residue inputs [6].

The BIO treatment primarily facilitated the enrichment of easily oxidized organic carbon (EOC) and dissolved organic carbon (DOC) within small macroaggregates (SMA) and MA, and significantly increased the DOC content in MA. This pattern may be partly related to the sorption of dissolved organic matter by surface functional groups, internal pores, and biochar–mineral interfaces of char particles, together with the relatively larger specific surface area of small-sized aggregates, which may provide additional interfaces for DOC and EOC redistribution [36,37,42]. In addition, the greater root volume and fork number observed under BIO may have expanded the spatial distribution of rhizodeposition, root exudates, sloughed cells, and fine-root residues, potentially contributing to labile carbon inputs in smaller structural domains [27]. However, because rhizodeposition, microbial assimilation, and carbon fluxes were not directly measured, the present data cannot distinguish whether the higher EOC and DOC mainly resulted from enhanced retention, greater production, slower turnover, or their combined effects [42]. The relatively low MBC content in the MA fraction under BIO suggests that the accumulated labile carbon was not proportionally reflected in microbial biomass. This may be related to the lower direct bioavailability of biochar-derived carbon, nutrient constraints, or differences in water, oxygen, and substrate accessibility across pore domains [29,41]. Recent evidence also indicates that warming can increase CO_2_ emissions from biochar-amended cropland soils, highlighting that biochar-associated increases in labile carbon should not be equated with greater net carbon retention under all environmental conditions [44].

In contrast, the BF treatment significantly increased the bulk-soil EOC content and the percentage of EOC in TOC, while exhibiting higher percentages of MBC in TOC within large macroaggregates (LMA) and SMA. Biochar-based fertilizer combines a carbonaceous matrix with mineral nutrients; the mineral fertilizer components may support root development and fresh carbon inputs, whereas the biochar component may delay the rapid loss of certain labile carbon fractions through surface adsorption and pore retention [42,43]. Therefore, the elevated EOC content under the BF treatment may reflect a readily turning-over labile carbon pool maintained by the combined effects of root-derived carbon inputs, nutrient supply, and retention at the char-particle interfaces. However, an increase in EOC and the percentage of EOC in TOC does not equate to an expansion of the stable organic carbon stock, particularly given that the total organic carbon (TOC) content under the BF treatment was relatively low, which can inherently inflate the percentage of EOC in TOC. Labile carbon must undergo microbial assimilation followed by aggregate occlusion or mineral surface binding to form more persistent organic carbon [24,38,45]. Consequently, the effect of the biochar-based fertilizer is more appropriately interpreted as an enhancement of the content and relative contribution of easily oxidized carbon, rather than a direct augmentation of the stable carbon stock.

### 4.4. Integrated Associations Among Pore–Aggregate Structure, Root Traits, and Labile Carbon Allocation

When integrating the outcomes from the various treatments, the results suggest that soil aggregates, pore networks, root traits, and labile organic carbon fractions do not function as mutually isolated indices, but may be linked within a reciprocal feedback framework. Aggregates constitute the solid skeletal framework of the soil, within and between which pore networks of diverse spatial scales are developed. The size distribution, connectivity, and directionality of these pores may influence the micro-scale spatial accessibility among soil moisture, oxygen, soluble substrates, and microorganisms. While actively responding to this pore environment, the root system continuously modifies the surrounding aggregate and pore architectures through physical penetration, compaction, rhizodeposition, exudate release, and root residue incorporation. Microbial utilization of exogenous organic matter and root-derived carbon may influence whether these inputs transiently persist as dissolved organic carbon (DOC) and easily oxidized organic carbon (EOC), are assimilated into microbial biomass carbon (MBC), or are further occluded within aggregate matrices and stabilized in mineral-associated carbon pools [36,37,38,45]. This feedback framework is conceptual and requires validation using temporal measurements and direct process indicators.

Given the limited sample size and the single post-treatment sampling event, the exploratory path analysis should be interpreted as a statistical synthesis of treatment-associated covariance rather than evidence of a sequential causal cascade. Nevertheless, this approach served as an exploratory framework for evaluating how soil physicochemical properties, aggregate structure, CT-resolved pore characteristics, root traits, and labile carbon fractions covaried under different amendment systems. The larger total association of soil physicochemical properties with labile carbon may be linked to combined variation in SOC, TN, pH, carbon-input quantity and quality, nutrient availability, and microbial responses [1,19,38]. Aggregate-related pathways may indicate possible associations with physical protection processes [46], whereas pore-related pathways may indicate possible associations with microbial microhabitat conditions and gas or water transport [47]. However, these potential mechanisms should be regarded as plausible interpretations rather than directly validated processes, because the CT analysis resolved only pores >30 μm and the model cannot conclusively separate the relative contributions of these alternative mechanisms.

Furthermore, root traits showed a negative net association with labile carbon in the structural equation model. This negative correlation should not be interpreted as root growth hindering soil fertilization, but may reflect the possibility that root development is inherently accompanied by concurrent, competing processes of carbon input and carbon consumption [48]. Although root exudation rates and root residue carbon components were not directly quantified in this study, the observed increases in peanut root length, root volume, and root surface area may have expanded the spatial footprint of rhizodeposition, root exudate release, and root residue incorporation. Concurrently, these root traits may also stimulate rhizosphere microbial metabolic activity, thereby accelerating the generation, utilization, and turnover of labile carbon fractions such as DOC and EOC. When fresh carbon inputs and microbial metabolic consumption are simultaneously accelerated, the net accumulation of labile carbon fractions at a specific sampling time point may not necessarily be positive [27,37]. These alternative processes cannot be separated with the present dataset. Differences in C:N:P inputs may also be related to microbial nutrient constraints and carbon-use strategies, making soil ecological stoichiometry a useful complementary context for interpreting carbon-pool changes [49]. BIO was associated with greater root morphological development and changes in labile-carbon pools, whereas BF was characterized by higher EOC accumulation and altered CT-resolved pore architecture. These patterns indicate differences in labile-carbon allocation among amendment systems, rather than direct evidence for enhanced long-term stable carbon sequestration. Although derived from a different ecosystem context, recent evidence on microbial-necromass accumulation provides a broader reminder that microbial-derived carbon should be measured directly before inferring long-term carbon persistence [50].

Several limitations should be considered. First, amendment type, nominal annual carbon input, C/N ratio, carbon chemistry, application rate, and nutrient-release dynamics were not independently controlled. Therefore, the results should be interpreted as contrasts among complete amendment systems rather than isolated amendment-type effects. Second, the number of independent microplots was limited (n = 12), and the correlation and SEM results should be regarded as exploratory. Third, CT resolved only pores > 30 μm under the present scanning conditions. Smaller pores, which may contribute to microbial habitat, water retention, nutrient diffusion, and carbon protection, were not characterized. Finally, measurements were conducted at a single sampling time, and several key processes, including soil penetration resistance, water availability, rhizodeposition, microbial processes, carbon mineralization, and persistent SOC fractions, were not directly measured. Future studies with repeated sampling, multi-resolution pore characterization, root–pore co-localization, microbial analyses, and direct measurements of carbon fluxes and persistent SOC fractions are needed to evaluate these proposed associations and mechanisms.

## 5. Conclusions

Under equivalent nitrogen, phosphorus, and potassium inputs, 15 years of four amendment systems were associated with distinct aggregate composition, CT-resolved (>30 μm) macropore architecture, peanut root traits, and labile-carbon allocation in this brown-soil peanut system. Pig manure (PMC) showed the highest CT-resolved total and connected porosities (19.01% and 10.86%) and the largest root surface area. Maize straw (CS) increased the proportion of large macroaggregates and produced the highest aggregate-size indices (MWD, 1.23 mm; GMD, 0.69 mm) and bulk-soil microbial biomass carbon (485.65 mg kg^−1^). In contrast, biochar (BIO) and biochar-based fertilizer (BF) were associated with greater CT-resolved macropore heterogeneity and differences in root length or root volume. BIO showed higher EOC and DOC contents in smaller aggregate fractions, whereas BF produced the highest bulk-soil EOC content (5.11 g kg^−1^) and percentage of EOC in TOC (52.45%), indicating a greater relative contribution of labile carbon within a comparatively low-TOC soil rather than an increase in the stable soil-carbon stock. These patterns reflect complete amendment systems that received equivalent N, P, and K inputs but differed in carbon input and amendment chemistry. Therefore, the findings should be interpreted as site-specific associations and hypotheses for further testing, rather than as evidence for pores < 30 μm or universal amendment recommendations.

## Figures and Tables

**Table 1:** Chemical properties and nominal annual carbon inputs of the organic resources used in the long-term field microplot experiment.

Organic Resources	C (%)	N (%)	P (%)	K (%)	C/N	Annual C Input (kg C ha^−1^ yr^−1^)
CS	44.4	1.01	0.09	0.77	44.4	1998
PMC	29.2	1.44	0.47	0.78	24.3	1168
BIO	33.3	0.50	0.37	0.49	66.6	500
BF	7.73	11.0	5.67	10.8	0.70	58

**Table 2:** Quantification of CT-resolved macropore structural parameters in intact soil cores.

Treatment	Isolated Porosity (%)	Pore Anisotropy (Dimensionless)	Mean Pore Diameter (mm)	Fractal Dimension (Dimensionless)
CS	0.73 ± 0.03 b	0.31 ± 0.02 b	0.35 ± 0.02 b	2.41 ± 0.03 b
PMC	0.65 ± 0.05 b	0.33 ± 0.01 b	0.31 ± 0.01 c	2.38 ± 0.01 b
BIO	1.05 ± 0.10 a	0.39 ± 0.02 a	0.42 ± 0.01 ab	2.49 ± 0.03 a
BF	0.98 ± 0.02 a	0.43 ± 0.01 a	0.46 ± 0.02 a	2.53 ± 0.02 a

Notes: Different lowercase letters in the table represent significant differences in different treatments at 0.05 level (*p* < 0.05, n = 3). Pore anisotropy and fractal dimension are dimensionless parameters. The same below.

**Table 3:** Pearson correlation coefficients (r) between peanut root traits and CT-resolved soil pore parameters.

Root Traits	Total Porosity	Connected Porosity	Isolated Porosity	Anisotropy	Mean Pore Diameter	Fractal Dimension
Root volume	−0.051	−0.400	0.556	0.645 *	0.393	0.449
Root length	−0.576 *	−0.623 *	0.710 **	0.911 **	0.716 **	0.750 **
Root branching	−0.096	−0.377	0.518	0.507	0.342	0.383
Root surface area	0.518	0.200	−0.015	0.228	−0.186	−0.118

Notes: Values are Pearson correlation coefficients (r; n = 12). ** indicates a highly significant correlation at *p* < 0.01, and * indicates a significant correlation at *p* < 0.05.

**Table 4:** Contents of soil labile organic carbon fractions and their proportions in total organic carbon (TOC).

Treatment	EOC (g kg^−1^)	MBC (mg kg^−1^)	DOC (mg kg^−1^)	EOC as Percentage of TOC (%)	MBC as Percentage of TOC (%)	DOC as Percentage of TOC (%)
CS	3.82 ± 0.03 b	485.65 ± 20.25 a	57.23 ± 1.28 a	36.68 ± 0.39 b	4.67 ± 0.19 a	0.55 ± 0.01 a
PMC	3.69 ± 0.10 b	402.70 ± 31.44 b	58.52 ± 3.66 a	35.58 ± 0.62 b	3.88 ± 0.29 ab	0.57 ± 0.03 a
BIO	4.04 ± 0.19 b	313.16 ± 24.48 bc	54.39 ± 3.52 a	41.12 ± 2.11 b	3.19 ± 0.25 bc	0.55 ± 0.03 a
BF	5.11 ± 0.25 a	249.34 ± 40.63 c	58.92 ± 3.09 a	52.45 ± 2.40 a	2.56 ± 0.43 c	0.61 ± 0.04 a

Notes: CS, PMC, BIO, and BF represent corn straw, pig manure compost, biochar, and biochar-based fertilizer treatments, respectively. Different lowercase letters indicate significant differences among treatments for the same variable at *p* < 0.05 (n = 3).

## Data Availability

The data that support the findings of this study are available from the authors upon reasonable request. The data are not publicly available because they are part of an ongoing long-term field experiment and will be used in subsequent analyses.

## Supplementary Materials

The following supporting information can be downloaded at: https://www.mdpi.com/article/10.3390/plants15172562/s1.
