# Supplementary material for: Long-Term Organic Amendment Systems Are Associated with Pore–Aggregate Structure, Root Traits, and Labile Organic Carbon Allocation in a Brown Soil
Source: Plants (Basel). 2026 Aug 23;15(17):2562. doi: 10.3390/plants15172562 (PMC13567501; doi:10.3390/plants15172562)
Supplement: Supplementary file 1 [file plants-15-02562-s001.zip › plants-4434443-supplementary.pdf]

Table S1. Basic physical and chemical properties of soil.

Treatment	Bulk Density（g/cm^3）	pH	SOC（g/kg）	TN（g/kg）	AK (g/kg)	AP (g/kg)
CS	1.23±0.03a	6.56±0.02b	10.49±0.12a	0.86±0.09b	24.56±0.17b	0.53±0.01a
PMC	1.14±0.05b	6.47±0.03c	10.56±0.10a	0.95±0.11a	24.78±0.24a	0.51±0.01a
BIO	1.19±0.04ab	6.76±0.03a	9.75±0.10b	0.79±0.10c	24.65±0.19ab	0.44±0.01b
BF	1.14±0.03b	6.65±0.02a	9.62±0.13b	0.78±0.09c	24.51±0.17b	0.41±0.01b

Note: Different lowercase letters in the table represent significant differences in different treatments at 0.05 level (P<0.05, n=3). The same below.

Table S2. Quantification of large pore structure in soil core.

Treatment	Isolated porosity (%)	Anisotropy	Mean pore diameter (mm)	Fractal dimension
CS	0.73±0.03b	0.31±0.02b	0.35±0.02b	2.41±0.03b
PMC	0.65±0.05b	0.33±0.01b	0.31±0.01c	2.38±0.01b
BIO	1.05±0.10a	0.39±0.02a	0.42±0.01ab	2.49±0.03a
BF	0.98±0.02a	0.43±0.01a	0.46±0.02a	2.53±0.02a

Note: Different lowercase letters in the table represent significant differences in different treatments at 0.05 level (P<0.05, n=3). The same below.

Table S3. Carbon quality of aggregates of different fractions under different treatments.

Treatment	Size	EOC/TOC（%）	MBC/TOC（%）	DOC/TOC（%）
CS	LMA（>2mm）	35.14±1.08c	1.94±0.03b	0.55±0.01b
	SMA（0.25-2mm）	40.85±2.20b	1.22±0.01b	0.60±0.03c
	MA（<0.25mm）	89.40±1.24b	2.32±0.04a	0.68±0.01d
PMC	LMA（>2mm）	41.617±0.25b	1.68±0.04c	0.61±0.02a
	SMA（0.25-2mm）	45.79±0.78a	1.23±0.03b	0.70±0.03a
	MA（<0.25mm）	92.31±2.62ab	1.17±0.02c	0.82±0.02c
BIO	LMA（>2mm）	41.32±2.35b	2.01±0.08b	0.55±0.02b
	SMA（0.25-2mm）	46.39±1.33a	1.37±0.02a	0.70±0.02a
	MA（<0.25mm）	96.03±2.04a	0.99±0.02d	0.96±0.01a
BF	LMA（>2mm）	44.46±2.10a	2.20±0.07a	0.55±0.01b
	SMA（0.25-2mm）	50.46±1.72a	1.43±0.02a	0.65±0.05b
	MA（<0.25mm）	92.15±1.79ab	1.35±0.02b	0.91±0.02b

Note: Different lowercase letters in the table represent significant differences in different treatments at 0.05 level ($P<0.05$, n=3). The same below.
